# Intelligence

**Published:** 1809-05

**Authors:** 


### INTELLIGENCE.

M. PULLY, a Neapolitan chemist, has recently analyzed the celebrated Dr. James's powder; and from his experiments on 19 grains, he states that he has found it to be composed of

| | |
|---|---|
| Oxide of antimony, at a maximum of oxydation | 7 |
| Phosphate of lime | 4 |
| Sulphate of potash | 4.5 |
| Free pot-ash, holding oxyd of antimony at a minimum | 3.5 |
| | 19. |

To recompose this powder, it is necessary, according to M. Pully, to take

| | |
|---|---|
| Sulphuret of antimony | 4 |
| Calcined phosphate of lime | 3 |
| Nitrate of potash | 8 |

These being powdered, mixed, and triturated together, are put into a crucible, which is to be covered and exposed to a strong heat, heat. During this operation, the oxygen of the nitric acid, attacking the sulphur of the antimonial sulphuret, converts it into sulphuric acid, which unites with a portion of the pot-ash, and forms sulphate of pot-ash. The remainder of the free pot-ash retains some antimony oxyded to a minimum. The white powder is the same as that sold by the name of Dr. James's. M. Pully asserts, that he has analyzed his powder, to compare it with the other, and has found it to contain the same principles, and in the same proportions.

Mr. W WELDON has analysed the water of a mineral spring, two miles to the south of Dudley, in Worcestershire, which has been famous from time immemorial, in the surrounding country, for its efficacy in various scrofulous and cutaneous diseases. In scrofula, in particular, it has been considered an almost infallible remedy. The spring flows into a well, about thirty-six feet in depth, and 7 ½ in diameter. The bottom is a ferruginous, argillaceous sandstone, through which is perforated a hole, whence the water issues, and rises to about four feet from the surface. The sides of the well near the top, are covered with a yellowish ochrey substance. When the water is fresh taken up, it is perfectly transparent and colourless. It is little refractive of light, nor can it be said to sparkle; but after standing for a short time, numerous small bubbles of air are seen adhering to the bottom and sides of the glass. After a time, it becomes rather turbid, and at length a pale ochreous precipitate falls down, leaving the water transparent. In large quantity, the water smells of sulphurated hydrogen, but if half a pint, or less, be examined, the odour is scarcely perceptible. The taste very much resembles sea-water. From a wine gallon, or 231 cubic inches were obtained:

| | |
|---|---|
| Of muriate of soda | 483 |
| lime | 311 |
| magnesia and alumina | 145 |
| iron | 26 |
| Of carbonate of iron | 9 |
| Of silica | 75 |
| Of earthy carbonates about | 45 |
| Of carbonic acid and sulphurated hydrogen, the latter in small proportion | 23,735 |
| Of azote | 12 |

Mr. PARKINSON has discovered in several species of marble, which he treated with muriatic or nitric acid, membraneous substances, which hung from the marble in light, flocculent, elastic membranes. These marbles were of a species formed by tubipores, madrapores, and corallites. In Kilkenny marble, the structure of the madrepores, and other testaceous substances which enter into its composition, is beautifully conspicuous, from the ground of the marble in which they are imbedded being of a deep black. This circumstance, in Mr. Parkinson's opinion, proves that two distinct lapidifying processes must have occurred in the formation of this marble; marble; and that its coralline, or testaceous part, had acquired a strong concretion previous to its being imbedded in the including mass of calcareous matter. A specimen of this marble, which Mr. Parkinson examined, in conformity with this opinion, exhibited no membranes when treated with diluted muriatic acid; but a black matter was deposited during the solution of the marble, which being dried and projected on melted nitre, immediately deflagrated; which circumstance shows the curious fact, that charcoal in substance entered into the composition of this marble. Mr. Parkinson supposes, that it must have been animal charcoal, from shells and corallines being visible in the marble. But this does not prove the absence of vegetable coal; nor is it, indeed, easy to determine the nature of the coaly substance, since we know that vegetable coal, lying in contact with animal substances, acquires all the characters of animal coal, sufficiently to be mistaken for it.

Mr. CARPUE will commence his Lectures on Anatomy, Surgery, &c. on the 1st. of June. Particulars may be known at Mr Carpue's, No. 50, Dean Street, Soho.

Dr. CLUTTERBUCK will commence his Summer Course of Lectures on the Theory and Practice of Physic, Materia Medica, and Chemistry, on Monday the 5th of June, at 9 o'clock in the morning, at his house, No. 1, Crescent, New Bridge Street, Blackfriars, where further Particulars may be had.

Dr. ADAMS having finished his "Inquiry into the Laws of Epidemics," is at leisure to complete his Edition of Mr. Hunter's Treatise on the Venereal Disease.

Mr. MARYAN, Surgeon, Rotherhithe Wall, will publish in the course of this month, a Treatise on Hydrophobia, explaining the impossibility of the disease being caused by the bite of any rabid animal.

